# Circulating Chemerin Levels in Obese and Non-obese Individuals and Its Association With Obesity in Metabolic Dysfunction-Associated Fatty Liver Disease

**DOI:** 10.7759/cureus.68105

**Published:** 2024-08-29

**Authors:** Aravindraj R, Renuka P, Vinodhini VM, Meenakshi Sundari SN

**Affiliations:** 1 Department of Biochemistry, SRM Medical College Hospital and Research Centre, Chengalpattu, IND; 2 Department of Internal Medicine, SRM Medical College Hospital and Research Centre, Chengalpattu, IND

**Keywords:** nafld, mafld, chemerin, insulin resistance, inflammation, obesity, physical activity

## Abstract

Introduction

The prevalence of obesity and related disorders is rapidly rising due to altered food habits, sedentary lifestyles and stress. Adipose tissue releases various hormones known as adipokines; one example is chemerin, which is primarily expressed by hepatocytes, adipocytes, and immune cells. Adipokine dysregulation in obesity initiates the cascade of inflammation and insulin resistance that leads to various metabolic disorders such as diabetes mellitus, metabolic syndrome (MS), and metabolic dysfunction-associated fatty liver disease (MAFLD).

Aim

The aim of our research is to determine serum chemerin levels in obese and non-obese individuals and to estimate the prevalence of MAFLD in obesity.

Materials and methods

This cross-sectional study was conducted at SRM Medical College Hospital & Research Centre, Tamil Nadu from August 2023 to December 2023. The study group comprised 45 obese and 45 non-obese individuals above 18 years of age. New MAFLD diagnostic criteria and FLI (Fatty Liver Index) formula were used to stratify the cohort. The Godin Leisure-Time Exercise questionnaire was used to assess physical activity levels. Visceral fat was assessed using a body composition analyzer. Student’s t-test and ANOVA were used to compare the difference in mean levels across the groups. Pearson’s correlation was used to correlate the analyzed parameters.

Results

Among our obese study participants, nearly 50% reported following a sedentary lifestyle. The prevalence of MAFLD in our obese study group was 44% whereas the prevalence of non-alcoholic fatty disease was found to be only 33%. Fasting plasma glucose (FPG), HbA1c, triglycerides (TG) and chemerin levels were found to vary significantly between the two groups. However, our study did not reveal the association of chemerin with MAFLD, BMI, or visceral fat in obesity. A significant difference in BMI, and visceral fat was observed across groups stratified by their physical activity levels assessed using the Godin leisure questionnaire.

Conclusion

Our study highlights the effect of physical activity on adipose tissue distribution and metabolic health and does not reveal any significant association of chemerin with MAFLD, BMI, or visceral fat in obesity. Nearly half of the studied obese individuals lead sedentary lifestyles, which highlights the importance of promoting physical activity in the prevention of obesity and related metabolic dysfunction. To validate these findings, future research should involve larger, diverse cohorts and include longitudinal data to track shifts in chemerin levels over time and their impact on metabolic health.

## Introduction

Chemerin, encoded by the retinoic acid receptor responder 2 gene, is secreted mainly from the liver, lungs and adipose tissue [1]. Apart from energy storage, adipose tissue, the largest endocrine gland synthesizes and secretes many adipokines. Chemerin is a potent adipokine that exhibits endocrine and paracrine functions through its receptor-chemokine-like receptor1 (CMKLR1) primarily expressed in adipocytes, hepatocytes and immune cells. Chemerin influences lipid and glucose metabolism apart from angiogenesis, adipogenesis, insulin release, inflammation and cytokine production [2].

The present population is at increased risk of developing obesity and diabetes due to high intake of processed foods, high-calorie diets, inactive lifestyle and stress [3]. In obesity, excess fat deposition causes adipose tissue inflammation and dysregulation of adipokines, leading to the pathogenesis of many obesity-related disorders [4].

Adipokine dysregulation linked to obesity and insulin resistance increases the risk of developing various metabolic disorders such as type 2 diabetes mellitus (T2DM), metabolic syndrome (MS), non-alcoholic fatty liver disease (NAFLD) and metabolic dysfunction-associated fatty liver disease (MAFLD) [5].

Compared to NAFLD's exclusion-based, liver-centric approach, MAFLD, which includes systemic metabolic dysfunction, offers a broader understanding of liver diseases [6]. MAFLD is associated with cardiovascular risk factors such as obesity, type 2 diabetes mellitus and dyslipidemia [7,8]. MAFLD is closely linked with the dysregulation of adipokines like chemerin [9]. The intricate relationship between obesity, chemerin levels and MAFLD highlights the complexity of metabolic disorders and the need for a better understanding of these interconnections between adipokines and metabolic disorders.

Thus, the aim of our research is to estimate the prevalence of MAFLD and serum chemerin levels in obese subjects. Furthermore, we aim to understand the association between chemerin, MAFLD, body fat distribution, and physical activity.

## Materials and methods

Study design and sample size

This analytical cross-sectional study was conducted at SRM Medical College Hospital and Research Centre in Chengalpattu, India, following the approval from the ethical committee (IEC no: SRMIEC-ST1122-233). Anthropometric measures were recorded after informed written consent was taken. The sample size was calculated as 45 based on the study of Gu et al. [10].

MAFLD diagnosis

MAFLD is diagnosed by detecting hepatic steatosis (presence of >5% fat in the liver) through ultrasound, together with the presence of one of three metabolic conditions: diabetes mellitus, overweight/obesity, or metabolic disorder (MD) [7]. For the Asian population, MD is diagnosed if any of the two following is present [7]: (1) waist circumference of 90/80 cm or more in men and women, respectively; (2) high blood pressure ≥130/85 mmHg or taking anti-hypertensive drugs; (3) plasma triglycerides (TG) of ≥150 mg/dl or under treatment; (4) plasma HDL-cholesterol, below 40 mg/dl for men and 50 mg/dl for women; (5) prediabetes, diagnosed by fasting glucose levels ≥100 mg/dl, two-hour post-prandial glucose ≥140 mg/dl, or HbA1c levels ≥5.7%; (6) increased homeostasis model assessment of insulin resistance (HOMA-IR) score (≥2.5) and (7) plasma high-sensitivity C-reactive protein (hs-CRP) level of >2 mg/L.

NAFLD diagnosis

NAFLD was diagnosed using the Fatty Liver Index (FLI) score; it was also used to quantify liver steatosis. Based on their FLI scores, an FLI score of 30 was considered NAFLD-free, and an FLI score >60 was considered NAFLD-positive. FLI is calculated by the following formula [11]:

$FLI = \frac{e^{0.953&times;loge(\text{triglycerides}) + 0.139&times;{BMI}+0.718&times;loge(\text{GGT}) + 0.053&times;{waist circumference}- 15.745}}{1 + e^{0.953&times;loge(\text{triglycerides}) + 0.139&times;{BMI} + 0.718&times;loge(\text{GGT}) + 0.053&times;{waist circumference}-15.745}} \times 100$

The Godin Leisure questionnaire was used to assess physical activity [12]. Body fat percentage was assessed using a body composition analyzer [13].

Inclusion and exclusion criteria

The study group comprised 90 participants above 18 years of age. Obese individuals (BMI ≥ 25 kg/m^2^) formed the obese group. Individuals with BMI in the range of 18-22.9 kg/m^2 ^formed the control group [7]. Pregnancy, thyroid ailments and chronic illness were considered as exclusion factors.

Blood sample collection

Under aseptic precautions, 5 ml of fasting venous blood sample was collected. The serum was stored at -80 C for estimation of chemerin by ELISA. Beckman Coulter auto analyzer was used to measure biochemical parameters such as FPG (mg/dL), HbA1c (%), cholesterol (mg/dL), TG (mg/dL), HDL-C and LDL-C (mg/dL). 

Statistical methods

Data was analyzed using SPSS Statistics v. 22.0 (IBM Corp., Armonk, NY). P-value < 0.05 was considered a significant difference. Continuous variables were presented as mean ± SD for normally distributed data. Student’s t-test and ANOVA were used to compare the difference in mean levels of analyzed biochemical parameters across the groups. Pearson’s correlation was used for correlating the analyzed biochemical parameters.

## Results

The obese group comprised 55% males and 45% females ranging in age from 25 to 65 years. The mean BMI of the obese and non-obese groups was found to be 27.67±2.8 and 20.5±1.5 kg/m2, respectively. The BMI in obesity ranged from 25 to 33.5 kg/m2. Table 1 shows fasting plasma glucose (FPG), HbA1c, triglycerides (TG), and chemerin levels were found to vary significantly between the two groups (Table 1).

**Table 1:** Student's t-test results comparing the various biochemical parameters among obese and non-obese groups. p < 0.05 indicates statistical significance. All values were expressed as mean ± SD. FPG: fasting plasma glucose; TG: triglycerides; HDL-C: high-density lipoprotein-cholesterol; LDL-C: low-density lipoprotein-cholesterol

Biochemical characteristics	Non-obese, n=45	Obese, n=45	P-value
FPG (mg/dL)	88.7±14	100±18	0.002*
HbA1c (%)	5±0.6	5.9±0.8	0.000*
Cholesterol (mg/dL)	173±36	179.5±33	0.417
TG (mg/dL)	93±56	121±38	0.008*
HDL-C (mg/dL)	46±13	41±8.7	0.051
LDL-C (mg/dL)	121±33	128±26.8	0.315
Chemerin (ng/mL )	1923.90±230.18	1315.33±112.6	0.02*

Table 2 shows the overall prevalence of MAFLD in our obese study population to be 44% (Table 2). 

**Table 2:** Prevalence of MAFLD among obese male and female individuals. MAFLD: metabolic associated fatty liver disease

Gender	MAFLD incidence; n (%)
Male	8 (18%)
Female	12 (26%)
Total	20 (44%)

Table 3 shows the overall prevalence of NAFLD in our obese study population as 33%. Males constituted 24% and females were 9% (Table 3).

**Table 3:** Prevalence of NAFLD among obese male and female individuals. NAFLD: non-alcoholic fatty liver disease

Gender	NAFLD incidence; n(%)
Male	11 (24%)
Female	4 (9%)
Total	15 (33%)

Table 4 shows that the MAFLD group showed higher chemerin values than the non-MAFLD group; however, there was no significant statistical difference in serum chemerin values between the two groups (Table 4).

**Table 4:** Student’s t-test results showing a comparison of chemerin between the obese MAFLD and the obese non-MAFLD groups. p < 0.05 indicates statistical significance. All values were expressed as mean ± SD. MAFLD: metabolic dysfunction-associated fatty liver disease; NAFLD: non-alcoholic fatty liver disease

Parameter	MAFLD (n=20)	Non-MAFLD (n=25)	P-value
Chemerin (ng/mL)	1473.8 ±208.4	1188.5±113.4	0.212

Table 5 shows chemerin did not show any significant correlation with BMI and visceral fat percentage in obesity. The mean chemerin levels in the study population were 1315.33 ± 112.6 ng/mL. The mean BMI was 27.67 ± 2.8 kg/m², and the mean visceral fat percentage was 15.7 ± 5.4%. The correlation analysis revealed that chemerin levels had a weak negative correlation with BMI (r = -0.103) and with visceral fat percentage (r = -0.185) (Table 5).

**Table 5:** Pearson’s correlation test showing the correlation of serum chemerin levels with BMI and visceral fat percentage in obesity. p < 0.05 indicates statistical significance. All values were expressed as mean ± SD.

Parameter	Pearson’s correlation	BMI (27.67±2.8 kg/m^2^)	Visceral fat % (15.7±5.4)
Chemerin (1315.33±112.6 ng/mL)	r-value	-0.103	-0.185
	p-value	0.501^ NS^	0.224

Table 6 shows obese individuals classified on the basis of physical activity assessed using the Godin leisure questionnaire showed a significant difference in BMI and visceral fat percentage. There is no significant difference in chemerin across the groups. Nearly half of the obese individuals reported a sedentary lifestyle followed by a moderately active group (40%) and an active group (11%) (Table 6).

**Table 6:** ANOVA results comparing serum chemerin, BMI and visceral fat in obese individuals classified on the basis of physical activity assessed using the Godin leisure questionnaire. p < 0.05 indicates statistical significance. All values were expressed as mean ± SD.

Parameters	Active group; n=5 (11%)	Moderately active group; n=18 (40%)	Sedentary group; n=22 (48%)	P-value
Chemerin (ng/mL)	1056.38±396.14	1553.39±942.81	1177.77±546.58	0.083
BMI (kg/m^2^)	26.06 ±0.74	26.79 ±1.56	28.76 ±3.45	0.024^*^
Visceral fat %	10.2±3.48	15.38±5.53	17.27±4.76	0.035^*^

## Discussion

The present study shows non-obese individuals exhibiting significant elevations in serum chemerin levels. However, analyses of chemerin levels by other researchers indicate elevations in obesity [14, 15]. The limited global research on chemerin levels and the scarcity of comprehensive studies might lead to discrepancies in findings across different populations. Therefore, our divergent results could stem from variations in sample demographics, genetic predispositions, or environmental factors.

Additionally, the multifaceted nature of the role of chemerin adds complexity to our interpretation [2]. Chemerin has been implicated in both pro-inflammatory and anti-inflammatory processes [16], contributing to the intricate mechanisms of its physiological effects. This is further complicated by individual variations in immune response and adipose tissue distribution [17].

Furthermore, the impact of unknown variables on the expression of chemerin cannot be ignored. Unidentified factors may complicate the suggested connection between chemerin and obesity, necessitating extensive research to better understand their contributions.

In our study, we found the prevalence of MAFLD among obese participants to be 44%. A meta-analysis by Xian et al. reports that MAFLD affects more than a third of the global population [18]. A subgroup analysis based on geographical areas by Chan et al. indicates that the highest prevalence of MAFLD is in Europe (55.33%), followed by Asia (36.31%) and the lowest is in North America (35.99%) [19]. MAFLD is closely associated with obesity, insulin resistance, and other metabolic disorders [5].

NAFLD ranges from simple steatosis to liver cirrhosis. Our study reports a NAFLD prevalence of 33% among obese adults. The prevalence of NAFLD in the Indian population was reported to be around 9% to 32%, varying across different geographic regions and populations [20].

NAFLD excludes patients with excessive alcohol consumption, viral hepatitis, and autoimmune conditions. But fatty liver diseases may coexist with the above-mentioned conditions and NAFLD thus may exclude those patients. On the other hand, the “inclusive” diagnostic criteria for MAFLD are based on the coexistence of hepatic steatosis and metabolic dysfunction. MAFLD can thus coexist with other liver diseases as well. This new MAFLD definition highlights the pathological significance of metabolic dysregulation in the onset and prognosis of fatty liver disease [7,18].

Nearly half of our obese participants manifested MAFLD, highlighting the risk of metabolic dysfunction associated with obesity. Given that obesity influences glucose metabolism, insulin resistance, fatty liver, and metabolic dysfunction [21], all of which are major contributing factors to MAFLD, addressing weight management through lifestyle changes such as healthy eating habits and increased physical activity can significantly improve metabolic health.

The higher prevalence of MAFLD among obese females compared to males can be attributed to several factors. Firstly, women tend to have higher visceral fat accumulation relative to men which contributes to fat deposition​ in the liver [22]. Secondly, estrogen plays a role in the prevention of fatty liver by enhancing lipid metabolism and reducing inflammation. However, estrogen levels decrease among post-menopausal women, increasing their risk for MAFLD [23]. Around 45% (n=9) of the women in our study were post-menopausal. Apart from total body fat, the fat distribution pattern, especially in the abdominal region, is a major risk factor for liver disorders [22]. Other factors such as genetic, metabolic differences, and physical activity levels may also contribute to the higher prevalence among females.

A comparison of chemerin between the MAFLD and non-MAFLD groups does not reveal any association of chemerin with MAFLD in obesity. Further research into the role of chemerin in the development of MAFLD is warranted given its involvement in inflammation and anti-inflammation [16].

In attempting to study the correlation of chemerin with BMI and visceral fat percentage using Pearson’s correlation analysis, we noted that there was no association. Visceral fat refers to the excessive accumulation of fat in the abdomen. This is associated with dysfunction of subcutaneous fat and abnormal storage of triglycerides in other parts of the body. Visceral obesity is directly linked to the presence of several risk factors for heart disease and metabolic disorders [24]. The findings of our study suggesting that chemerin levels are not associated with either BMI or visceral fat percentage in our obese population is unexpected. Shin et al. have suggested potential links between chemerin and obesity and visceral fat [25].

Notably, nearly 50% of our obese study participants report following a sedentary lifestyle with a relatively smaller proportion being actively involved in physical activities; similar lifestyle patterns were noted in a study by Anjana et al. who reported 54% of people in the southern states of India were categorized as inactive [26].

The ANOVA test was used to assess the variations in MAFLD risk parameters among groups stratified by levels of physical activity assessed using the Godin Leisure-time exercise questionnaire. The results indicate a significant variation among the groups in terms of BMI and visceral fat percentage. These findings suggest that physical activity influences anthropometric and metabolic parameters. Physically active people tend to have lower weights, BMI values, and visceral fat percentages compared to those who are less active.

Visceral fat dysfunction is suggested as a major factor in causing and sustaining chronic inflammation, liver steatosis, and eventual systemic insulin resistance. This further leads to the development of metabolic disorders and associated complications [27]. Inflamed adipocytes release proinflammatory cytokines, both in the immediate vicinity and throughout the body. These cytokines interfere with the normal functioning of adipose tissue itself, as well as the other organs [16].

The significant differences in visceral fat observed across groups stratified by physical activity levels suggest that engaging in regular physical activity may have a profound effect on body composition and metabolic health. Weight, BMI, and visceral fat percentage are crucial indicators of overall health and are closely linked to the risk of developing chronic diseases such as cardiovascular disease, diabetes, and metabolic syndrome [28].

The lack of significance while comparing serum chemerin levels across active, moderate, and sedentary groups indicates that the level of physical activity might not have a substantial impact on chemerin concentrations among the participants in our study. On the other hand, Lloyd et al. suggest a potential effect of acute exercise in decreasing circulating chemerin levels in obese individuals [29]. Though physical activity is known to impact various aspects of metabolic health, its specific effect on chemerin levels remains unclear.

The limitations noted are the absence of an evaluation of MAFLD among the control population since metabolic dysfunction is reported among lean people also and the limited sample size.

## Conclusions

Metabolic dysfunction associated with fatty liver disease is prevalent in nearly 50% of the obese study population. Our study does not reveal an association of chemerin with MAFLD, BMI and visceral fat in obesity. Further research with a larger sample size is needed to confirm these relationships. Obese individuals stratified on the basis of physical activity showed a significant difference in BMI and visceral fat. Nearly half of the studied obese individuals lead sedentary lifestyles, highlighting the importance of promoting physical activity as a crucial component in the prevention and management of obesity and related metabolic dysfunction. MAFLD may be better suited for identifying at-risk individuals compared to the NAFLD criteria.
